# Supplementary material for: Antimicrobial and antioxidant activities of neem assisted silver-modified zeolite X synthesized from kaolin
Source: PLoS One. 2026 Feb 13;21(2):e0343110. doi: 10.1371/journal.pone.0343110 (PMC12904421; doi:10.1371/journal.pone.0343110)
Supplement: S1 File — (DOCX) [file pone.0343110.s001.docx]

| SAMPLE | VALUE | %INHIBI | VALUE | %INHIBI | VALUE | %INHIBI | MEAN | STDEV |
| --- | --- | --- | --- | --- | --- | --- | --- | --- |
| ABTS | 1.5 |  | 1.5 |  | 1.5 |  |  |  |
| Ag-ZEO X (0.05) | 0.696 | 53.6 | 0.754 | 49.73333 | 0.638 | 57.46667 | 53.6 | 3.866667 |
| Ag-ZEO X (0.10) | 0.747 | 50.2 | 0.676 | 54.93333 | 0.619 | 58.73333 | 54.62222 | 4.275165 |
| Ag-ZEOX (0.15) | 0.619 | 58.73333 | 0.804 | 46.4 | 0.589 | 60.73333 | 55.28889 | 7.762684 |
| Ag-ZEO X (0.2) | 0.544 | 63.73333 | 0.613 | 59.13333 | 0.558 | 62.8 | 61.88889 | 2.431582 |
| Ag-ZEO X (0.25) | 0.483 | 67.8 | 0.521 | 65.26667 | 0.48 | 68 | 67.02222 | 1.523641 |
|  | | | | | | | | |
| SAMPLE | VALUE | %INHIBI | VALUE | %INHIBI | VALUE | %INHIBI | MEAN | SDV |
| ABTS | 1.5 |  | 1.5 |  | 1.5 |  |  |  |
| ZEO X (0.05) | 1.032 | 31.2 | 1.308 | 12.8 | 1.412 | 5.866667 | 16.62222 | 13.09204 |
| ZEO X (0.1) | 1.012 | 32.53333 | 0.927 | 38.2 | 0.667 | 55.53333 | 42.08889 | 11.98301 |
| ZEO X (0.15) | 0.798 | 46.8 | 0.757 | 49.53333 | 0.888 | 40.8 | 45.71111 | 4.46733 |
| ZEO X (0.2) | 0.575 | 61.66667 | 0.687 | 54.2 | 0.592 | 60.53333 | 58.8 | 4.023818 |
| ZEO X (0.25) | 0.616 | 58.93333 | 0.568 | 62.13333 | 0.539 | 64.06667 | 61.71111 | 2.592582 |
|  | | | | | | | | |

| SAMPLE | VALUES | %IHBI | VALUES | %INBI | VALUES | %INHIBI | MEAN | SDV |
| --- | --- | --- | --- | --- | --- | --- | --- | --- |
| ABTS | 1.5 |  | 1.5 |  | 1.5 |  |  |  |
| VIT C (0.25) | 0.096 | 98.6010 | 0.028 | 98.1101 | 0.030 | 98.0202 | 98.2438 | 0.31 |
| VIT C (0.20) | 0.040 | 97.3011 | 0.036 | 97.6210 | 0.0031 | 97.9501 | 97.6241 | 0.32 |
| VIT C (0.15) | 0.042 | 97.2110 | 0.040 | 97.3211 | 0.039 | 97.4301 | 97.3207 | 0.06 |
| VIT C (0.10) | 0.055 | 96.3412 | 0.047 | 96.8411 | 0.041 | 97.2401 | 96.8075 | 0.40 |
| VIT C (0.05) | 0.070 | 95.3311 | 0.054 | 96.4302 | 0.037 | 97.5401 | 96.4338 | 1.11 |

| SAMPLE | VALUE | %IHBI | VALUES | %INBI | VALUES | %INHIBI | MEAN | SDV |
| --- | --- | --- | --- | --- | --- | --- | --- | --- |
| DPPH | 0.746 |  | 0.746 |  | 0.746 |  |  |  |
| VIT C (0.5) | 0.133 | 82.2212 | 0.125 | 83.3104 | 0.120 | 84.4512 | 83.9943 | 1.12 |
| VIT C (0.1) | 0.132 | 82.3201 | 0.110 | 85.3211 | 0.108 | 85.5202 | 84.3871 | 1.72 |
| VIT C (0.15) | 0.118 | 84.1203 | 0.102 | 86.3204 | 0.086 | 88.5201 | 86.3203 | 2.20 |
| VIT C (0.2) | 0.108 | 85.5210 | 0.100 | 86.6301 | 0.095 | 87.7311 | 86.6271 | 1.19 |
| VIT C (0.25) | 0.097 | 87.0001 | 0.138 | 87.5011 | 0.090 | 88.0001 | 87.5004 | 0.51 |

| SAMPLE | VALUE | %IHBI | VALUES | %INBI | VALUES | %INHIBI | MEAN | SDV |
| --- | --- | --- | --- | --- | --- | --- | --- | --- |
| DPPH | 0.746 |  | 0.746 |  | 0.746 |  |  |  |
| Ag-ZEO X (0.25) | 0.318 | 57.37265 | 0.355 | 52.41287 | 0.277 | 62.86863 | 57.55139 | 5.230173 |
| Ag-ZEOX (0.2) | 0.377 | 49.46381 | 0.393 | 47.31903 | 0.439 | 41.15282 | 45.97855 | 4.314605 |
| Ag-ZEO X (0.15) | 0.45 | 39.67828 | 0.45 | 39.67828 | 0.495 | 33.64611 | 37.66756 | 3.482676 |
| Ag-ZEO X (0.10) | 0.571 | 23.45845 | 0.576 | 22.7882 | 0.571 | 23.45845 | 23.23503 | 0.386964 |
| Ag-ZEO X (0.05) | 0.654 | 12.33244 | 0.664 | 10.99196 | 0.656 | 12.06434 | 11.79625 | 0.709317 |
|  | | | | | | | | |
| SAMPLE | values | %IHBI | VALUES | %INBI | VALUES | %INHIBI | MEAN | SDV |
| DPPH | 0.746 |  |  |  |  |  |  |  |
| ZEO X (0.25) | 0.474 | 36.46113 | 0.448 | 39.94638 | 0.474 | 36.46113 | 37.62288 | 2.012213 |
| ZEO X (0.20) | 0.577 | 22.65416 | 0.502 | 32.70777 | 0.545 | 26.9437 | 27.43521 | 5.0448 |
| ZEO X (0.15) | 0.599 | 19.70509 | 0.6 | 19.57105 | 0.599 | 19.70509 | 19.66041 | 0.077393 |
| ZEO X (0.10) | 0.633 | 15.14745 | 0.633 | 15.14745 | 0.63 | 15.5496 | 15.2815 | 0.232178 |
| ZEO X (0.05) | 0.601 | 19.437 | 0.655 | 12.19839 | 0.655 | 12.19839 | 14.61126 | 4.179211 |
