# Supplementary material for: Antimicrobial and antioxidant activities of neem assisted silver-modified zeolite X synthesized from kaolin
Source: PLoS One. 2026 Feb 13;21(2):e0343110. doi: 10.1371/journal.pone.0343110 (PMC12904421; doi:10.1371/journal.pone.0343110)
Supplement: Attachment — Submitted filename: Response to Reviewers.docx [file pone.0343110.s002.docx]

**Response to Reviewers**

**Manuscript Title:**
**Antimicrobial and Antioxidant Activities of Neem-Assisted Silver-Modified Zeolite X Synthesized from Kaolin**

**Manuscript ID:** [PONE-D-25-58884]

We thank the Editor and Reviewers for their constructive comments, which have greatly improved the clarity, scientific rigor, and presentation of our manuscript. All comments have been carefully addressed, and the manuscript has been extensively revised accordingly. A tracked-changes version and a clean revised manuscript have been submitted as requested.

Below, we provide a point-by-point response to all reviewer comments.

**Reviewer #1**

**Comment 1**

*The introduction lacks a novelty statement. The last paragraph should focus on the aim and novelty of the work.*

**Response:**
We thank the reviewer for this valuable comment. The Introduction has been revised to clearly articulate the novelty and scientific contribution of the study. A new paragraph has been added at the end of the Introduction highlighting (i) the use of neem-mediated green synthesis, (ii) integration of Ag into kaolin-derived zeolite X, and (iii) simultaneous evaluation of antimicrobial and antioxidant properties.
**(Revised manuscript: Introduction, final paragraph)**

**Comment 2**

*Ensure consistent terminology for silver throughout the manuscript.*

**Response:**
The manuscript has been thoroughly edited to ensure consistent terminology. “Silver,” “Ag⁺,” and “Ag-zeolite X” are now used consistently throughout the text depending on chemical context.

**Comment 3**

*Correct scientific naming of microorganisms.*

**Response:**
All microbial names have been corrected and standardized according to international nomenclature (italicized genus and species on first mention).
**(Revised throughout the manuscript)**

**Comment 4**

*Remove repetition of DPPH and ABTS descriptions.*

**Response:**
Redundant descriptions of DPPH and ABTS assays were removed, and the methodology was streamlined for clarity.
**(Methods section: Antioxidant activity)**

**Comment 5**

*Provide accurate temperature and processing details for metakaolin preparation.*

**Response:**
Detailed calcination conditions (temperature, duration, and heating procedure) have been added in the Materials and Methods section under “Synthesis of Zeolite X.”

**Comment 6**

*Use correct botanical nomenclature for neem.*

**Response:**
The scientific name *Azadirachta indica* has been used consistently at first mention and appropriately thereafter throughout the manuscript.

**Comment 7**

*Expand the description of characterization techniques.*

**Response:**
The descriptions of XRD, FTIR, SEM, and Raman spectroscopy have been significantly expanded, including instrumental conditions, scan ranges, and analytical rationale.

**Comment 8**

*Clarify preparation of stock solutions in MIC assays.*

**Response:**
The preparation of stock solutions, dilution steps, and concentrations have been clarified and standardized in (MIC determination).

**Comment 9**

*Include JCPDS card numbers and literature support for XRD peaks.*

**Response:**
Relevant JCPDS reference numbers and supporting literature have been added to the XRD results and discussion.

**Comment 10**

*Add more peak assignments in FTIR and Raman analyses.*

**Response:**
Additional FTIR and Raman peak assignments have been incorporated with corresponding literature citations, and interpretations were expanded to explain bonding and framework changes.

**Comment 11**

*Improve Table 1 formatting and link antimicrobial results to SEM findings.*

**Response:**
Table 1 has been reformatted for clarity. The antimicrobial results are now explicitly correlated with SEM-observed surface morphology and particle aggregation.

**Comment 12**

*Correct p-value formatting.*

**Response:**
All statistical values have been reformatted according to journal standards (e.g., *p* < 0.05).

**Reviewer #2**

**Comment 1**

*Revise abstract to include key results.*

**Response:**
The abstract has been rewritten to clearly summarize synthesis, characterization, antimicrobial and antioxidant outcomes, and key findings.

**Comment 2**

*Strengthen novelty and justification in the Introduction.*

**Response:**
The Introduction has been reorganized to emphasize the novelty of neem-assisted silver modification of zeolite X and its dual antimicrobial–antioxidant functionality.

**Comment 3**

*Ensure uniformity in chemical and biological nomenclature.*

**Response:**
All chemical names, microbial names, and abbreviations have been standardized throughout the manuscript.

**Comment 4**

*Provide detailed characterization methodology.*

**Response:**
Expanded experimental descriptions have been added for XRD, SEM, FTIR, Raman, and antimicrobial testing.

**Comment 5**

*Include experimental temperature and time parameters.*

**Response:**
All experimental conditions, including temperatures, durations, and concentrations, are now explicitly stated.

**Comment 6**

*Strengthen discussion with literature support.*

**Response:**
The Results and Discussion section has been substantially expanded with comparisons to relevant literature and mechanistic interpretations.

**Comment 7**

*Add peak labeling in figures and scale bars in SEM images.*

**Response:**
All figures have been updated with appropriate labels, peak assignments, and scale bars.

**Comment** 8

*Include antimicrobial images in main manuscript.*

**Response:**
Representative antimicrobial assay images have been included in the revised manuscript.

Comment 9

*Clarify the novelty of the study.*

**Response:**
The novelty is now explicitly stated in the Abstract, Introduction, and Discussion, emphasizing the green synthesis route, synergistic antimicrobial–antioxidant behaviour, and application relevance.
